# Supplementary material for: An Influenza A Vaccine Based on the Extracellular Domain of Matrix 2 Protein Protects BALB/C Mice Against H1N1 and H3N2
Source: Vaccines (Basel). 2019 Aug 19;7(3):91. doi: 10.3390/vaccines7030091 (PMC6789677; doi:10.3390/vaccines7030091)
Supplement: Supplementary file 1 [file vaccines-07-00091-s001.pdf]

Supplementary Material

Table 1: Sickness scores allocation

Point	Symptoms
0	Healthy
1	Ruffled fur
1	Hunched back posture
1	Reduced activity
2	Severely reduced activity
